# Key Hepatokines Linking MASLD and Type 2 Diabetes: From Pathophysiological Mechanisms to Therapeutic Modulation

**DOI:** 10.3390/ijms27177917

**Published:** 2026-09-04

**Authors:** Georgeta Liliana Foia, Ancuta Goriuc, Ionut Luchian, Dana Gabriela Budala, Vasilica Toma, Maria Bogdan, Bogdan Minea, Larisa Ghemiș

**Affiliations:** 1 Grigore T. Popa University of Medicine and Pharmacy Iasi, 700115 Iasi, Romania; georgeta.foia@umfiasi.ro (G.L.F.); ionut.luchian@umfiasi.ro (I.L.); vasilica.toma@umfiasi.ro (V.T.); bogdan-minea@umfiasi.ro (B.M.); larisa.danaila@umfiasi.ro (L.G.); 2 University of Medicine and Pharmacy, 200349 Craiova, Romania; maria.bogdan@umfcv.ro

**Keywords:** type 2 diabetes mellitus, MASLD, metabolic disorder, hepatokines, FGF21, fetuin-A, LECT2, selenoprotein P, ANGPTLs

## Abstract

Type 2 diabetes mellitus (T2D) is a major and growing global health burden that is associated with substantial cardiovascular, renal, and metabolic complications and is increasingly recognized as a systemic disorder involving multiple organs, particularly the liver. Metabolic dysfunction-associated steatotic liver disease (MASLD) affects more than half of individuals with T2D, and the two conditions have a bidirectional relationship: T2D promotes the development and progression of MASLD, including advanced fibrosis and increased liver-related mortality, while MASLD worsens insulin resistance and metabolic control, favoring the onset of T2D. In this context, the liver is increasingly viewed not only as a metabolic organ but also as an endocrine one, secreting hepatokines that act on distant tissues to regulate insulin sensitivity, inflammation, glucose metabolism, and lipid homeostasis. Through these actions, hepatokines are thought to represent one of the mechanistic links between MASLD and T2D. This narrative review summarizes current evidence on several of the most extensively studied hepatokines, including fibroblast growth factor 21, fetuin-A, fetuin-B, leukocyte cell-derived chemotaxin-2, selenoprotein P, angiopoietin-like proteins, and retinol-binding protein 4. As a distinctive feature, the review also discusses emerging pharmacological strategies targeting hepatokine pathways and evaluates how commonly used antidiabetic therapies may modulate hepatokine secretion.

## 1. Introduction

Diabetes mellitus is a metabolic disorder characterized by chronic hyperglycemia, resulting from defects in insulin action, insulin secretion, or both, leading to reduced peripheral glucose uptake and/or increased hepatic glucose production through enhanced gluconeogenesis and inappropriate glycogenolysis [1,2]. In clinical practice, the diagnosis is based on standardized criteria (fasting plasma glucose, oral glucose tolerance test, glycated hemoglobin (HbA1c)), with type 2 diabetes mellitus (T2D) being the predominant phenotype [1]. T2D is a pathology of major interest, as it represents a continuously increasing global burden, with a substantial impact on both mortality and healthcare system costs. Recent data estimate that, currently, 589 million adults aged 20–79 years worldwide are living with diabetes, a number estimated to rise to 853 million by 2050 [3]. The increase in prevalence is particularly alarming, given that T2D is closely associated with a wide spectrum of micro- and macrovascular complications. These include cardiovascular, renal, ocular and diabetic foot diseases, and represent the leading cause of morbidity and mortality among these patients [4,5,6].

T2D is no longer viewed solely as a pancreas-mediated glucose homeostasis impairment, but rather as a systemic disease involving multiple organs [6]. Meta-analytic data highlight that non-alcoholic fatty liver disease (NAFLD), now redefined as metabolic dysfunction-associated steatotic liver disease (MASLD), affects more than half of individuals with T2D globally [7,8]. Hence, hepatic dysfunction in T2D is increasingly recognized not as a simple comorbidity but rather as an active pathophysiological contributor that can magnify insulin resistance, systemic inflammation, and lipoprotein dysfunction, thereby increasing the risk of cardiovascular and renal complications [7,9].

The relationship between T2D and MASLD is currently considered bidirectional. Meta-analyses indicate that patients with T2D have a substantially increased risk of developing MASLD, with an estimated global prevalence of approximately 55.5% among patients with T2D [8]. Moreover, T2D accelerates the progression of MASLD toward advanced liver fibrosis and is associated with increased liver-related and all-cause mortality [10]. Conversely, patients with MASLD have a higher risk of developing T2D and of worsening metabolic control [11]. An important conceptual shift is the move from viewing the liver merely as an affected organ, particularly in the context of T2D, toward recognizing it as an endocrine and secretory organ that directly influences systemic metabolism. Hepatokines are an example of liver-secreted proteins, capable of regulating systemic metabolic processes by modulating insulin sensitivity in peripheral tissues such as skeletal muscle and adipose tissue, inflammation, and lipid metabolism [12,13]. Among them, fibroblast growth factor (FGF21), fetuin-A, fetuin-B, leukocyte cell-derived chemotaxin-2 (LECT2), selenoprotein P (SeP), angiopoietin-like proteins (ANGPTLs), and retinol-binding protein 4 (RBP4) have received increasing attention for their potential involvement in the pathophysiological link between MASLD and T2D.

This narrative review aims to synthesize the latest findings, highlighting the relationship between MASLD and T2D, with particular emphasis on the role of selected key hepatokines within this framework. In addition, emerging therapeutic strategies targeting hepatokine pathways, as well as the influence of anti-diabetic drugs on hepatokine regulation and MASLD progression, will be addressed.

## 2. Methodology

The purpose of this narrative review is to integrate current evidence on the pathophysiological mechanisms and the therapeutic modulation of key hepatokines involved in the bidirectional relationship between MASLD and T2D. This review focuses on FGF21, fetuin-A, fetuin-B, LECT2, SeP, ANGPTLs, and RBP4. A literature search of major databases, including PubMed/MEDLINE, Web of Science, and Scopus, was supplemented by searches in Google Scholar and Google Search. Keywords such as “MASLD”, “NAFLD”, “MASH”, “NASH”, “metabolic disorder”, “type 2 diabetes”, “insulin resistance”, and “hepatokine”, including the names of the selected hepatokines and other relevant terms, were used in various combinations to identify relevant studies. The search focused on recent studies; however, a restriction on publication date was not included.

We considered for inclusion original research, in vitro, animal and human studies, clinical trials, prior reviews and meta-analyses published in peer-reviewed, English-language journals. Given the narrative nature of this review, articles were selected based on their relevance to the topic and the quality of the evidence they provided, rather than according to a predefined systematic protocol.

## 3. MASLD and Type 2 Diabetes: A Bidirectional Pathophysiological Relationship

The term non-alcoholic fatty liver disease (NAFLD) has been replaced by metabolic dysfunction-associated steatotic liver disease (MASLD). According to the updated definition, MASLD is diagnosed when liver steatosis is present, confirmed by imaging or histology, together with at least one of five cardiometabolic risk factors and in the absence of chronic alcohol consumption [14,15]. The MASLD spectrum ranges from solely steatosis to metabolic dysfunction-associated steatohepatitis (MASH), previously known as nonalcoholic steatohepatitis (NASH), and covers the progress to fibrosis, cirrhosis, and MASH-related hepatocellular carcinoma (HCC) [16,17].

Recent studies emphasize that NAFLD/MASLD should not be regarded only as a hepatic manifestation of metabolic dysfunction, but rather as an active contributor to systemic metabolic impairment. Hepatic lipid accumulation, lipotoxicity, insulin resistance, gut dysbiosis and chronic low-grade inflammation interact in a complex manner, fostering both liver injury and extrahepatic metabolic complications [18,19,20]. In a meta-analysis, MASLD was significantly associated with a higher risk of developing both T2D and metabolic syndrome over approximately five years of follow-up [21]. MASLD worsens both hepatic and peripheral insulin resistance, contributes to atherogenic dyslipidemia, and promotes the systemic release of inflammatory cytokines and hepatokines, thereby facilitating the development of T2D [22].

On the other hand, among patients with T2D, MASLD is highly prevalent on a global scale, affecting about 65% of this population, with prevalence increasing over time and reaching the highest levels in Eastern Europe and the Middle East [23]. In patients with T2D, insulin resistance may promote hepatic fat accumulation by increasing adipose tissue lipolysis and the delivery of free fatty acids to the liver, while hyperinsulinemia and hyperglycemia stimulate hepatic de novo lipogenesis [24,25,26,27]. These processes contribute to lipotoxicity, oxidative stress, mitochondrial dysfunction and inflammatory activation, creating a favorable environment for progression from simple steatosis to steatohepatitis and fibrosis [28,29,30,31].

Moreover, the link between MASLD and T2D is also supported by recent genetic findings. A cross-trait meta-analysis identified 115 genes shared by the two conditions, some being involved in lipid, phospholipid and glycerophospholipid metabolism. Additional differential gene expression analysis confirmed that 15 core genes were associated with both MASLD and T2D and were correlated with inflammation and carcinoma cells [32]. Therefore, the relationship between MASLD and T2D appears to be bidirectional, with diabetes availing for the progression and severity of liver disease, while MASLD itself contributes to worsening glucose metabolism and cardiometabolic risk.

### Pro- and Anti-Inflammatory Cytokines in MASLD and T2D

Chronic low-grade inflammation represents another important link between MASLD and T2D [33]. Obesity and T2D are characterized by systemic immune dysregulation, including dysregulated cytokine and chemokine levels, alterations in leukocyte numbers and activation states, apoptosis, and tissue fibrosis [34]. Within the liver, inflammatory signals, including cytokines and adipocytokines derived from immune and other cell types, further contribute to insulin resistance and the shared pathophysiology of T2D and MASLD [35]. Accordingly, both MASLD and MASH are characterized by chronic, low-grade hepatic inflammation, driven by various cytokines with pro- and anti-inflammatory properties. Among them, interleukin-10 (IL-10), IL-22, IL-25, and IL-27 generally exert protective effects, whereas tumor necrosis factor-alpha (TNF-α), IL-2, IL-6, IL-18, IL-33, and interferons (IFNs) appear to have dual effects, depending on the stage of MASLD [36]. The clinical relevance of these pathways is supported by a meta-analysis of 51 studies involving 36,074 patients and 47,052 controls, which found that elevated C-reactive protein (CRP), IL-1β, IL-6, TNF-α, and intercellular adhesion molecule-1 (ICAM-1) levels were significantly associated with MASLD, while CRP, IL-1β, and TNF-α were also associated with MASH and hepatic fibrosis [37]. Taken together, these findings suggest that cytokine imbalance contributes to both metabolic dysfunction and liver disease progression and that selected inflammatory mediators may serve as biomarkers of disease presence and severity.

## 4. Key Hepatokines Linking MASLD and Type 2 Diabetes

**Figure 1:**
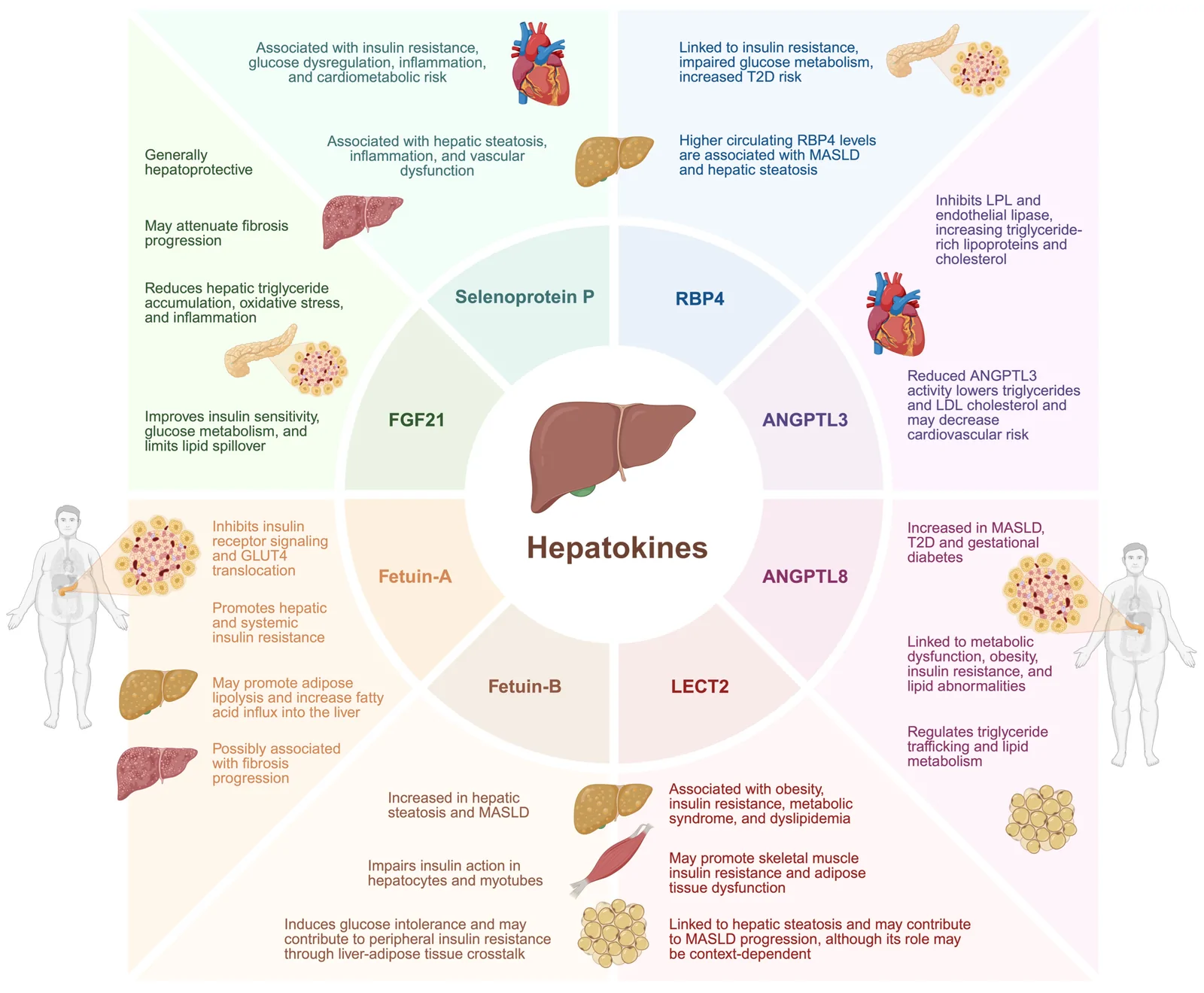
Key hepatokines linking MASLD and Type 2 diabetes. Created in BioRender. Ghemis, L. (2026) https://BioRender.com/e9c7d4p.

Hepatokines are liver-derived secretory proteins that act as important mediators of inter-organ communication and contribute to the regulation of systemic metabolic homeostasis. Through endocrine, paracrine, and autocrine mechanisms, hepatokines influence key biological processes such as glucose and lipid metabolism, insulin signaling, inflammation, oxidative stress, mitochondrial function, and fibrogenesis. Their production and circulating levels may change in response to nutritional status, hepatic lipid accumulation, metabolic stress, and chronic inflammation. As a result, hepatokines have gained increasing attention as potential biomarkers of metabolic impairment and as possible therapeutic targets. Among the most studied hepatokines, fibroblast growth factor 21 (FGF21), fetuin-A, fetuin-B, leukocyte cell-derived chemotaxin-2 (LECT2), selenoprotein P (SeP), angiopoietin-like proteins (ANGPTLs), and retinol-binding protein 4 (RBP4) appear to be most relevant in mediating communication between the liver and peripheral tissues [38,39,40,41,42,43]. An overview of the major hepatokines linking MASLD and T2D is presented in Table 1 and Figure 1.

### 4.1. Fibroblast Growth Factor 21 (FGF21)

#### 4.1.1. General Considerations

**Figure 2:**
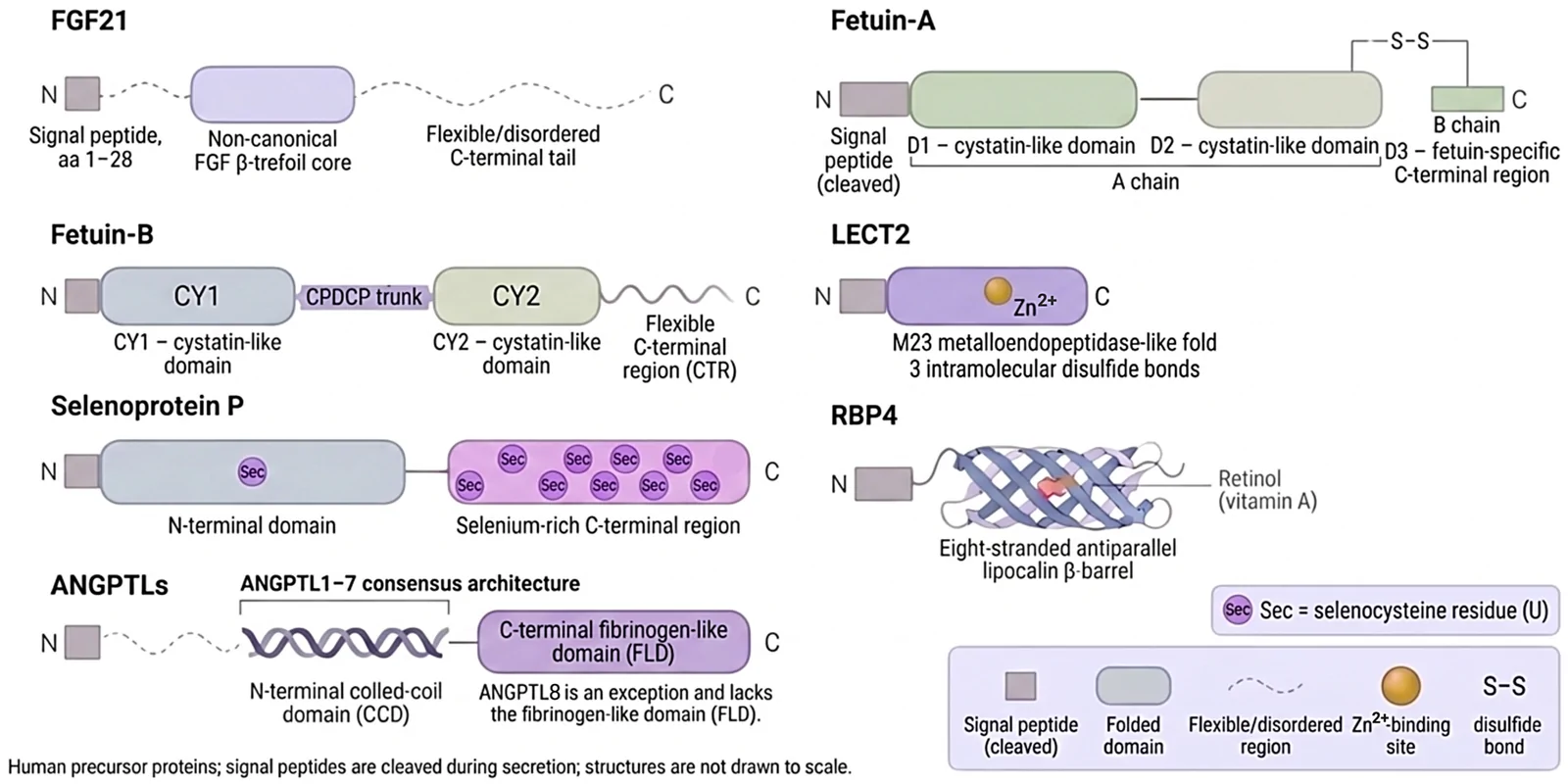
Schematic representation of the structures of selected human hepatokines: FGF21—fibroblast growth factor 21, fetuin-A, fetuin-B, LECT2—leukocyte cell-derived chemotaxin-2, selenoprotein P, ANGPTLs—angiopoietin-like proteins, and RBP4—retinol-binding protein 4. Created in BioRender. Ghemis, L. (2026) https://BioRender.com/vuzra5a.

The human FGF21 is a protein encoded by the *FGF21* gene located on chromosome 19. It is synthesized as a 209-amino acid precursor containing an N-terminal signal peptide, which is cleaved to produce the mature 181-amino acid protein [123]. Structurally, mature FGF21 consists of a non-canonical FGF β-trefoil core followed by a flexible, intrinsically disordered C-terminal region (Figure 2) [124]. Human and mouse FGF21 share approximately 75% amino acid sequence identity, and experimental animal studies support its role as an important metabolic regulator [123]. Under physiological conditions, circulating FGF21 is primarily derived from the liver in both mice and humans [44]. FGF21 acts through fibroblast growth factor receptors (FGFRs), which must form heterodimers with the co-receptor β-klotho to activate downstream signaling [125]. The FGFR family consists of four receptor tyrosine kinases: FGFR1, FGFR2, FGFR3, and FGFR4, with FGF21 showing the highest affinity for FGFR1 [13]. FGF21 binding to FGFR1 in the presence of β-klotho triggers FGFR1 autophosphorylation and subsequent activation of the mitogen-activated protein kinase (MAPK) pathway, leading to extracellular signal-regulated kinases 1/2 (ERK1/2) phosphorylation [126].

The restricted expression of β-klotho in metabolically active tissues, including liver, adipose tissue, brain, and pancreas, explains why FGF21 exerts its metabolic effects mainly in these target organs [45,46]. In this context, FGF21 has been shown to improve insulin sensitivity, reduce hepatic steatosis and oxidative stress, regulate glucose metabolism, and promote fatty acid oxidation through activation of adenosine monophosphate-activated protein kinase (AMPK) and histone protein deacetylase Sirtuin 1 (SIRT1) pathways [47]. Overall, FGF21’s downstream signaling network is complex and interconnected, involving AMPK, mTOR, the hypothalamic–pituitary–adrenal axis, glucose transporter 1 (GLUT1), with indirect effects on c-Jun N-terminal kinase (JNK), and nuclear factor kappa-light-chain-enhancer of activated B cells (NF-κB) pathways [13]. As a key regulator of lipid metabolism and energy balance, FGF21 emerged as a major focus in the development of therapeutic agents targeting hypertriglyceridemia and MASLD [48].

#### 4.1.2. FGF21 in MASLD and T2D

In the context of T2D, the metabolic actions of FGF21 are considered largely protective, as they may improve insulin sensitivity by reducing glucotoxicity and limiting lipid spillover, two processes actively implicated in T2D progression [48,49]. However, circulating FGF21 levels are often elevated in obesity, insulin resistance, and T2D. This paradox has been interpreted as a state of “FGF21 resistance,” in which target tissues become less responsive to FGF21 signaling. Consequently, the liver may increase FGF21 production as a compensatory response to metabolic stress, although the protective effect becomes insufficient [49,50]. Although elevated circulating FGF21 levels may reflect metabolic stress and disease severity in T2D and MASLD, FGF21 is not yet established as a routine diagnostic biomarker.

Circulating FGF21 levels increase with body mass index (BMI) and are elevated in MASLD and MASH, reflecting hepatic metabolic stress [127]. In a murine study, Kim et al. showed that FGF21 is induced during endoplasmic reticulum stress through the PKR-like ER kinase–eukaryotic translation factor 2α–activating transcription factor 4 (PERK-eIF2α-ATF4) pathway and may attenuate hepatic lipid accumulation, liver injury, and metabolic deterioration [128]. Thus, in MASLD, FGF21 is generally considered hepatoprotective. Additionally, it reduces hepatic triglyceride accumulation by promoting fatty acid oxidation and suppressing de novo lipogenesis [48,50]. One of the mechanisms by which FGF21 can reduce hepatic steatosis is a decrease in the flux of free fatty acids from adipose tissue to the liver, mediated by its interaction with its principal receptor, FGFR1c, expressed in adipose tissue. In addition, FGF21 inhibits hepatic lipogenesis, curtails caloric intake, modulates iron metabolism, and enhances insulin sensitivity in both adipose and muscle tissue, thereby favoring glucose uptake in peripheral organs [51]. It also improves mitochondrial function and reduces oxidative stress, which are essential mechanisms in preventing hepatocyte injury [48,50]. In a murine model of D-galactose-induced liver injury, FGF21 administration attenuated the D-galactose-induced increases in reactive oxygen species (ROS) and malondialdehyde (MDA), restored hepatic glutathione (GSH) levels, and enhanced the activities of superoxide dismutase (SOD), catalase (CAT), and glutathione peroxidase (GSH-Px). These effects were accompanied by increased nuclear abundance of nuclear factor erythroid 2-related factor 2 (Nrf2) and upregulation of Nrf2-regulated antioxidant genes, whereas activation of the phosphoinositide 3-kinase/protein kinase B (PI3K/Akt) signaling pathway was associated with reduced hepatocyte apoptosis [129]. In other experimental models, FGF21 attenuated hepatic fibrogenesis through complementary mechanisms, including reduced hepatic lipid accumulation and macrophage activation [52], and inhibition of hepatic stellate cell activation via suppression of the TGF-β/Smad2/3 and NF-κB signaling pathways [53]. These mechanisms explain why FGF21 analogs are currently being investigated for MASH and fibrotic MASLD. Recent reviews of FGF21 agonists report improvements in liver fat content, metabolic parameters and markers of liver injury, although the degree of fibrosis amelioration remains an active area of investigation [130,131,132].

### 4.2. Fetuin Family

#### 4.2.1. General Considerations

Fetuins are proteins belonging to the cystatin superfamily and are structurally related to cysteine protease inhibitors, characterized by conserved cysteine residues involved in protease inhibitory activity. The fetuin family consists of two paralogue proteins, encoded by duplicated genes, fetuin-A and fetuin-B [133].

Fetuin-A, also known as α2-Heremans-Schmid glycoprotein, is a multifunctional serum protein encoded by the *AHSG* gene located on chromosome 3q27. Structurally, fetuin-A consists of two polypeptide chains linked by disulfide bonds and organized into three domains: two cystatin domains, D1 and D2, and a C-terminal specific region (Figure 2) [134]. Fetuin-A is primarily produced and secreted by the liver, although extrahepatic sources, including visceral and subcutaneous adipose tissue, have also been described [55,56]. Functionally, fetuin-A may play important roles in the human body, including insulin signaling, inflammation, mineral and protein metabolism, and being involved in the maintenance of hepatic and systemic metabolic homeostasis [135,136,137].

Fetuin-B is the second member of the fetuin family and shares structural similarities with fetuin-A. It consists of two tandem cystatin-like domains, CY1 and CY2, connected by a bonding region containing a characteristic “CPDCP-trunk”, followed by a C-terminal region with no regular secondary structure (Figure 2) [68]. Fetuin-B is produced mainly by hepatocytes and has been described as a secreted hepatocyte-derived factor linking hepatic steatosis and impaired glucose metabolism [39].

#### 4.2.2. Fetuin-A in MASLD and T2D

In the context of T2D, fetuin-A acts as an important hepatokine connecting hepatic metabolic dysfunction with systemic insulin resistance. By inhibiting insulin receptor autophosphorylation and downstream insulin signaling, fetuin-A suppresses insulin-stimulated glucose transporter 4 (GLUT4) translocation, reducing glucose uptake in peripheral skeletal muscle [57,58]. Over time, these effects may contribute to reduced insulin sensitivity and impaired glucose tolerance, thereby increasing the risk of progression toward T2D [59,60]. Additionally, fetuin-A may amplify metabolic inflammation, particularly in the presence of elevated free fatty acids, by activating Toll-like receptor 4 (TLR4) and promoting NF-κB signaling. The resulting boost in proinflammatory cytokines, including TNF-α and IL-6, may further worsen insulin resistance and contribute to β-cell stress over time [54,61,62]. Data on oxidative stress and fetuin-A are limited, with the available evidence derived from cohorts of patients with gynecological conditions. Women with polycystic ovary syndrome (PCOS), recently renamed polyendocrine metabolic ovarian syndrome (PMOS), exhibited elevated fetuin-A levels and an altered oxidative stress profile compared with controls. However, fetuin-A was not significantly correlated with total antioxidant status, total oxidant status, or the oxidative stress index [138].

Regarding metabolic dysfunction, meta-analyses have consistently shown that higher circulating fetuin-A levels are associated with T2D. Roshanzamir et al. reported significantly higher fetuin-A concentrations in patients with T2D than in controls and found that elevated fetuin-A was associated with increased T2D risk, while Guo et al. showed that each standard-deviation (SD) increase in circulating fetuin-A was associated with a 23% higher risk of incident T2D [63,64]. This association was further supported by Sujana et al., who reported a comparable 24% higher risk of incident T2D for each 1-SD increase in fetuin-A, independently of subclinical inflammation, adiponectin, and liver fat content [65]. Moreover, a case–control study in individuals without MASLD further showed that fetuin-A levels were already higher in those with impaired glucose tolerance and newly diagnosed T2D, suggesting that fetuin-A may be involved early in glucose dysregulation, independently of cardiometabolic risk factors [60].

In MASLD, hepatic lipid overload may stimulate fetuin-A expression and secretion. Elevated fetuin-A can then exacerbate hepatic and systemic insulin resistance, triggering adipose tissue lipolysis and increasing free fatty acid delivery to the liver. This creates a vicious cycle in which hepatic fat accumulation enhances fetuin-A release, while fetuin-A further promotes steatosis through insulin resistance and increased lipid influx [50,54,56]. Fetuin-A may also contribute to the inflammatory progression of MASLD, particularly in the presence of elevated free fatty acids, by activating TLR4/NF-κB signaling and promoting cytokine release [40,66]. This pro-inflammatory state may favor the transition from simple steatosis to steatohepatitis and contribute subsequently to a profibrogenic microenvironment. Consequently, circulating fetuin-A has been associated with liver and vascular fibrosis-related markers in patients with MASLD, suggesting that fetuin-A may be considered a potential biomarker for predicting the progression of hepatic and vascular fibrosis in the affected individuals [54,67]. Based on these findings, fetuin-A has been proposed as a non-invasive biomarker for MASLD diagnosis and severity assessment, with one 2024 study reporting 82% sensitivity, 90% specificity, and 86% accuracy at a cut-off value > 702.5. However, these results require validation in larger, independent cohorts before fetuin-A can be considered for routine clinical use [139].

#### 4.2.3. Fetuin-B in MASLD and T2D

Fetuin-B is a liver-derived glycoprotein that has been proposed as a hepatokine linking hepatic steatosis with impaired glucose metabolism. Experimental and clinical data suggest that fetuin-B expression and secretion increase proportionally with hepatic lipid accumulation, and higher circulating fetuin-B levels have been reported in individuals with hepatic steatosis and T2D [39,69]. Fetuin-B has been shown to impair insulin action in hepatocytes and myotubes and to induce glucose intolerance in mice, while its silencing in obese mice improved glucose tolerance [39]. Fetuin-B may also aggravate oxidative stress, as its overexpression in HepG2 cells significantly increased intracellular ROS and MDA production while decreasing SOD and GSH levels [140]. More recently, fetuin-B has also been linked to liver–adipose tissue crosstalk, as circulating fetuin-B was associated with white adipose tissue genes involved in cytokine/chemokine and insulin signaling pathways, suggesting a possible role in peripheral insulin resistance [70].

In adult patients, higher circulating fetuin-B levels have been linked to hepatic steatosis, HOMA-IR (Homeostatic Model Assessment for Insulin Resistance), skeletal muscle insulin sensitivity, and free fatty acid suppression during hyperinsulinemic–euglycemic clamp, suggesting that fetuin-B may participate in the crosstalk between the liver and adipose tissue [71]. Fetuin-B levels in white adipose tissue were strongly associated with peripheral insulin resistance in both mice and humans. Moreover, fetuin-B promoted a pro-inflammatory response in adipocytes, which may contribute to the development of peripheral insulin resistance [72]. Meex et al. showed that fetuin-B links hepatic steatosis with impaired glucose metabolism, while Pasmans et al. further suggested that fetuin-B may contribute to peripheral insulin resistance by promoting adipose tissue inflammation [39,72]. In a large meta-analysis of 30 studies, including 3800 patients with MASLD and 3614 controls, circulating fetuin-B levels were significantly higher in patients with MASLD than in controls [73]. In addition, recent meta-analytic data support the inclusion of fetuin-B among hepatokines associated with MASLD-related metabolic dysfunction, although its clinical relevance remains less clearly established compared to fetuin-A or FGF21 [42].

Overall, current evidence suggests that fetuin-B may act as a steatosis-responsive hepatokine involved in both MASLD and T2D. Therefore, fetuin-B appears to be a promising, but still insufficiently characterized mediator and potential biomarker of hepatic and systemic metabolic impairment.

### 4.3. Leukocyte Cell-Derived Chemotaxin-2 (LECT2)

#### 4.3.1. General Considerations

Leukocyte cell-derived chemotaxin-2 (LECT2), initially described as neutrophil chemotactic factor, is a multifunctional secreted protein mainly produced by hepatocytes. LECT2 is a 16kDa secreted protein, composed of 151 amino acids stabilized by 3 intramolecular disulfide bonds [74,75,76]. Structurally, LECT2 adopts an M23 metalloendopeptidase fold with a conserved Zn(II)-binding site but lacks the catalytic histidine and has a substrate-binding groove obstructed by an N-terminal loop (Figure 2) [141]. LECT2, encoded by the human *LECT2* gene, is primarily expressed in adult and fetal hepatocytes and is subsequently secreted into the circulation. Beyond its chemotactic functions, LECT2 is involved in the regulation of inflammation and immune responses [74,75,76].

#### 4.3.2. LECT2, MASLD and T2D

In T2D, LECT2 has emerged as a hepatokine closely related to insulin resistance, obesity, and broader metabolic dysfunction. Circulating LECT2 concentrations were reported to be significantly higher in newly diagnosed patients with T2D compared to controls, with further elevation in obese individuals with T2D, and were independently associated with metabolic parameters such as HOMA-IR, fasting insulin, BMI, triglycerides, and HDL-C levels [75]. These findings are consistent with experimental evidence showing that LECT2 promotes skeletal muscle insulin resistance in obesity, suggesting a potential mechanistic role in the development of impaired glucose metabolism [77]. More recent data further support this concept, as patients with T2D and metabolic syndrome showed higher LECT2 levels than those without metabolic syndrome, and LECT2 remained independently associated with metabolic syndrome after adjustment for confounding factors [78]. Beyond its hepatic origin, LECT2 may also contribute to adipose tissue dysfunction, adipose tissue LECT2 expression and circulating LECT2 levels being increased in obesity and associated with insulin resistance [79].

However, the relationship between LECT2 shift and metabolic improvement appears complex, as studies in patients undergoing bariatric surgery have reported either no significant postoperative change in circulating LECT2 levels in T2D patients, or associations between LECT2 changes and reductions in fat mass and NASH scores after laparoscopic sleeve gastrectomy [80,81]. Moreover, acute glucose loading was shown to decrease circulating LECT2 levels independently of glucose tolerance status, suggesting that LECT2 regulation may depend on both chronic metabolic stress and short-term nutritional signals [82]. Overall, current evidence suggests that LECT2 may participate in the pathophysiology of T2D by linking hepatic and adipose tissue dysfunction with insulin resistance, dyslipidemia, and metabolic syndrome, although its behavior may vary according to disease stage, obesity status, and metabolic interventions.

In MASLD, LECT2 appears to be closely linked to hepatic lipid accumulation, inflammation, and metabolic dysfunction. Clinical data showed that circulating LECT2 levels were significantly higher in individuals with MASLD than in those without this condition and were associated with metabolic syndrome, obesity-related parameters, lipid profiles, high-sensitivity C-reactive protein (hsCRP), and liver aminotransferase levels, suggesting that the relationship between LECT2 and MASLD may be partly mediated by abdominal obesity and altered lipid metabolism [83]. At the hepatic level, LECT2 expression is increased in steatotic and inflamed liver tissue and correlates with BMI and inflammatory markers such as C-C chemokine receptor type 2 (CCR2) and TLR4, supporting its role as a hepatokine that links hepatic steatosis to inflammation. A study that combined analyses of human liver biopsy samples with in vivo experiments in high-fat-diet-fed Lect2-knockout mice and in vitro studies using a murine Kupffer cell line concluded that increased LECT2 promotes liver inflammation by shifting liver-resident macrophages toward a pro-inflammatory M1-like phenotype [84]. In a separate murine model of hepatic ischemia–reperfusion injury, *Lect2* silencing significantly reduced hepatic ROS levels and the expression of IL-1β, IL-6, and TNF-α [142]. LECT2 has been shown to act through several pathways involved in MASLD progression, including STAT-1 signaling, ER stress/UPR–ATF4-mediated transcriptional regulation, and JNK/mTOR/SREBP-1-related lipogenic and insulin-resistance pathways [85,86,87]. However, the role of LECT2 in MASLD may be context-dependent, since LECT2 deletion aggravated steatosis and altered macrophage activation in a bacterial translocation-mediated MASLD/MASH model, suggesting that its effects may vary according to inflammatory triggers and specific disease context [88]. Taken together, LECT2 may function as a steatosis-sensing hepatokine involved in the transition from simple lipid accumulation to inflammatory MASLD, although its precise role appears complex and requires further clarification.

### 4.4. Selenoprotein P (SeP)

#### 4.4.1. General Considerations

The human selenoprotein family comprises 25 molecules, including Selenoprotein P (SELENOP, SelP, SePP, SePP1, SeP). SeP contains 10 selenocysteine residues and 17 cysteine and 23 histidine residues, being considered the main selenium transport protein in mammals [89,90,91]. Structurally, human SeP contains one selenocysteine residue in its N-terminal redox motif and nine additional residues clustered within the selenium-rich C-terminal region (Figure 2) [143,144]. SeP has been studied in different pathologies, including cancers and metabolic-derived conditions such as T2D and MASLD [92,145]. In overweight and obese patients, plasma/serum levels of selenium and SeP were not significantly altered, but a meta-analysis highlighted a reduced glutathione peroxidase activity, suggesting impaired antioxidant defense [146]. However, circulating SeP levels were increased in patients with impaired glucose metabolism and were associated with several cardiometabolic features, including insulin resistance, inflammation, and atherosclerosis [92].

#### 4.4.2. Selenoprotein P, MASLD and T2D

SeP has been implicated in T2D as a hepatokine associated with glucose dysregulation, insulin resistance, and cardiometabolic risk. Circulating SeP levels were reported to be higher in patients with T2D or prediabetes than in individuals with normal glucose tolerance and were positively associated with BMI, waist circumference, triglycerides, glucose, HbA1c, inflammatory markers, and insulin resistance [92]. SeP may also be relevant to diabetic complications, as a recent study reported a non-significant tendency toward higher SeP levels with increasing retinopathy severity [93]. However, SeP regulation appears complex, as oral glucose loading acutely reduced circulating SeP independently of glucose tolerance status, and EPA-related changes in SeP (performed by eicosapentaenoic acid—EPA) did not fully explain improvements in hepatic insulin sensitivity [82,94]. Overall, current evidence suggests that SeP may contribute to T2D-related metabolic impairment and may have biomarker potential, although its regulation and clinical relevance remain to be fully elucidated.

In MASLD, selenoprotein P has been linked to hepatic steatosis, insulin resistance, inflammation and fibrosis, although evidence remains partly conflicting. Several clinical studies reported higher circulating SeP levels in individuals with MASLD, with associations with visceral adiposity, liver enzymes, hsCRP, insulin resistance, altered lipid metabolism, vascular dysfunction and hepatic fibrosis [95,96,97]. Experimental data further suggest that SeP may aggravate hepatic triglyceride accumulation through inhibition of the AMPK/ACC pathway [98]. However, other studies reported lower SeP levels in definite MASH, and lifestyle-induced reductions in hepatic steatosis were not accompanied by significant SeP changes [99,100]. A 2026 prospective case–control study found reduced SeP values in MASLD, with the lowest levels in significant fibrosis, suggesting that low SeP may reflect depleted antioxidant capacity in progressive disease [101]. Overall, SeP appears to be a metabolically relevant yet complex hepatokine in MASLD, whose clinical significance may vary depending on disease stage, fibrosis severity, selenium status, and metabolic phenotype.

### 4.5. ANGPTL (Angiopoietin-like Proteins) Family

#### 4.5.1. General Considerations

ANGPTLs constitute a family of proteins with a structure similar to that of angiopoietin. The literature describes eight ANGPTLs, numbered from 1 to 8, which are involved in numerous physiological and pathophysiological processes, including carbohydrate and lipid metabolism, hematopoiesis, inflammation and neoplastic shift [102]. These processes are regulated through binding to different receptors, depending on the cell type, including LILRB2 (Leukocyte Immunoglobulin-Like Receptor B2) and PirB (Paired Immunoglobulin-Like Receptor B), receptors for ANGPTL2, ANGPTL3, and ANGPTL5 in hematopoietic stem cells, as well as LILRB3, the receptor for ANGPTL8 in cardiomyocytes. In addition, ANGPTLs can also interact with various integrins [103]. Although ANGPTL1-7 have a structure similar to that of angiopoietins, with a N-terminal coiled-coil domain (CCD) and a C-terminal fibrinogen-like domain (FLD), they do not bind to the Tie1 and Tie2 receptors due to the absence of a cysteine-rich motif in the FLD. In contrast, ANGPTL8 has a truncated structure and lacks the FLD (Figure 2) [103,104]. ANGPTL3, ANGPTL4, and ANGPTL8 primarily regulate triglyceride metabolism by inhibiting lipoprotein lipase (LPL), the enzyme responsible for the hydrolyzation of circulating triglycerides. Fasting-induced ANGPTL4 reduces adipose uptake of triglyceride-derived fatty acids by promoting LPL unfolding, cleavage, and subsequent degradation, whereas ANGPTL8 opposes this effect after feeding, thereby preserving LPL activity [147]. ANGPTL3 and the complex between ANGPTL3 and ANGPTL8 (ANGPTL3/8) inhibit LPL activity in oxidative tissues by interacting with sequences surrounding LPL’s catalytic pocket, using the proximal regions of its N-terminal α-helices. This interaction triggers unfolding of LPL’s α/β-hydrolase domain, resulting in irreversible loss of its catalytic activity [148]. Beyond their role in triglyceride metabolism, ANGPTL molecules exhibit multiple biological functions, being involved in several human pathologies such as cancer, vascular disorders, chronic inflammation and metabolic impairments [105,106,107].

#### 4.5.2. ANGPTLs, MASLD and T2D

ANGPTL3, ANGPTL4 and ANGPTL8 are key regulators of lipoprotein metabolism, mainly through their ability to modulate lipoprotein lipase activity. By controlling the hydrolysis of triglyceride-rich lipoproteins, they influence how circulating fatty acids are delivered to different tissues. ANGPTL3 is primarily produced by the liver and acts together with ANGPTL8 to regulate triglyceride metabolism in the fed state, while ANGPTL4 is more closely linked to fasting and helps limit lipid uptake in certain tissues [149]. However, the role of ANGPTL4 in lipid metabolism was studied in a meta-analysis that found no association between circulating ANGPTL4 concentrations and triglyceride levels or coronary heart disease risk [108]. At the tissue level, preclinical studies showed that hepatocyte-specific loss of ANGPTL4 enhances fatty acid uptake, thereby increasing fatty acid oxidation, ROS generation, and AMPK activation [150].

ANGPTL3 has been identified as an important regulator of plasma lipid metabolism and has gained attention as a potential therapeutic target in situations characterized by atherogenic dyslipidemia, including T2D. Being produced primarily in the liver, ANGPTL3 modulates lipid handling by inhibiting key lipolytic enzymes such as lipoprotein lipase and endothelial lipase, thereby influencing the circulating levels of triglyceride-rich lipoproteins and cholesterol fractions [151]. Human genetic studies have consistently shown that naturally occurring loss-of-function variants in ANGPTL3 are associated with reduced plasma triglycerides and LDL cholesterol, as well as a lower risk of cardiovascular disease, supporting its causal involvement in lipid homeostasis [152].

On the opposite, ANGPTL 8, also known as betatrophin, is consistently associated with metabolic dysfunction, as meta-analysis findings show. Adults with MASLD recorded higher circulating ANGPTL8 concentrations than controls, hence supporting its potential role as a biomarker of metabolic liver dysfunction and as a possible indicator for monitoring disease progress [109,110]. Elevated ANGPTL8 circulating levels have also been reported in obese subjects compared with nonobese individuals, although heterogeneity across studies was present due to glycemic status [111]. Similarly, increased levels of ANGPTL8 were noticed in polycystic ovary syndrome, regardless of BMI, with higher levels particularly associated with insulin resistance and positive correlations with age, free androgen index, and free testosterone, suggesting a possible link between ANGPTL8 and both metabolic and endocrine dysfunctions [112]. ANGPTL8 concentrations were also significantly elevated in women with gestational diabetes mellitus, especially during the third trimester and among women with BMI ≥ 28 kg/m^2^ [113]. Consequently, in T2D, circulating ANGPTL8 was generally higher in subjects with T2D compared with healthy controls, although subgroup analyses suggest that this association may vary according to ethnicity and obesity status [114,115,116]. A 2025 updated meta-analysis supports higher ANGPTL8 levels in MASLD, but large prospective studies are still needed to prove diagnostic or prognostic value [110].

Overall, these findings indicate that ANGPTL8 may represent a promising biomarker and potential therapeutic target in metabolic disorders. However, the large heterogeneity reported in several meta-analyses highlights the need for consistent, prospective studies to clarify its contribution to these conditions.

### 4.6. Retinol-Binding Protein 4 (RBP4)

#### 4.6.1. General Considerations

Retinol-binding protein 4 (RBP4) is a 21 kDa protein secreted predominantly by the liver and adipose tissue. It was originally identified as the main circulating transport protein for retinol, also known as vitamin A, and other retinoid derivatives [117]. Structurally, RBP4 belongs to the lipocalin family and consists of an eight-stranded antiparallel β-barrel with an internal hydrophobic ligand-binding cavity and a single α-helix, enabling the binding and transport of retinol (Figure 2) [153].

#### 4.6.2. RBP4, MASLD and T2D

In 2017, Chen et al. investigated the association between circulating RBP4 levels and MASLD in a community-based cohort of 2938 Chinese adults [118]. The study found that individuals with MASLD had significantly higher serum RBP4 concentrations than those without hepatic steatosis, and this association remained significant after adjustment for metabolic and anthropometric confounders. Elevated RBP4 levels were also positively correlated with obesity, insulin resistance, and other components of the metabolic syndrome, suggesting a potential role of RBP4 in MASLD pathogenesis. However, due to its cross-sectional design, the study could not establish causality and did not specifically evaluate patients with histologically confirmed MASH [118]. Another study conducted by OuYang et al. pointed out that serum vitamin A and RBP4 levels were significantly higher in MASLD subjects than in healthy controls and increased progressively with disease severity. These findings suggest that alterations in the vitamin A–RBP4 axis are associated with MASLD progression and may have potential value as non-invasive biomarkers of disease severity [119]. An experimental study combining in vitro experiments in Kupffer cells and hepatocytes with in vivo experiments in high-fat-diet-fed mice showed that exosomal RBP4, primarily derived from hepatocytes, promoted M1-like Kupffer cell polarization through the NOX2/ROS/NF-κB signaling pathway, thereby increasing TNF-α production and hepatic lipid accumulation [154].

Elevated RBP4 levels have been consistently associated with insulin resistance, impaired glucose metabolism, and an increased risk of T2D, although some variability exists according to sex, renal function, metabolic status, and measurement methods [120]. Experimental and clinical evidence suggests that RBP4 can disrupt insulin signaling in skeletal muscle, liver, and adipose tissue, while more recent studies highlight its potential direct contribution to pancreatic β-cell dysfunction through activation of the stimulated by retinoic acid 6-Janus kinase 2/signal transducer and activator of transcription 1 (STRA6-JAK2/STAT1) pathway, leading thus to reduced insulin synthesis and secretion [121,122,155]. RBP4 seems, thus, to represent a promising biomarker and potential therapeutic target in T2D, but further translational and interventional studies are required before RBP4-lowering approaches can be translated into clinical practice.

### 4.7. Heterogeneity of Hepatokines in Patients with MASLD and T2D

Because MASLD and T2D are complex diseases, circulating hepatokine levels may vary even within the same patient subgroup. This variability is illustrated by Stefan et al., who performed a cluster analysis of 185 individuals at high risk of developing T2D. The analysis identified three phenotypes, two of which had similar levels of liver fat and insulin resistance but different adiponectin and fetuin-A levels [50]. Yamasandhi et al. showed that serum fetuin-A levels were elevated in patients with T2D and MASLD but were not associated with HbA1c or markers of advanced fibrosis [156]. Similarly, in a study of 270 patients with T2D, El-Ashmawy and Ahmed found that circulating fetuin-B levels were higher in patients with moderate-to-severe hepatic steatosis than in those without MASLD. By contrast, RBP4 levels did not differ according to MASLD severity within the same cohort [157]. Regarding heterogeneity in treatment response, an illustrative example is provided by Li et al., who studied 20 overweight patients with newly diagnosed T2D and MASLD, all of whom were treated with liraglutide. FGF21 levels decreased significantly only in patients who achieved a reduction of at least 29% in liver fat content [158]. Taken together, current evidence suggests that hepatokine profiles are heterogeneous among patients with coexisting T2D and MASLD, with this variability appearing to be related primarily to the severity of hepatic involvement and treatment response. Future studies should simultaneously assess a broader panel of hepatokines within a single well-characterized cohort of patients with coexisting MASLD and T2D, as this approach could identify distinct hepatokine profiles, better characterize interindividual heterogeneity, and reduce variability arising from differences between study populations and diagnostic methods.

## 5. Therapeutic Modulation of Hepatokines in MASLD and Type 2 Diabetes

The management of MASLD associated with T2D remains focused on weight reduction through lifestyle modification, but it can be complemented by pharmacological agents such as glucagon-like peptide-1 receptor agonists (GLP-1 RAs) or sodium–glucose cotransporter 2 (SGLT2) inhibitors. However, although these agents have substantial effects on reducing hepatic fat, improving glycemic control and slowing the progression of fibrosis, their efficacy declines considerably in advanced stages of hepatic fibrosis and cirrhosis, which underscores the need for new therapeutic targets [159]. In this context, hepatokines have attracted considerable interest, as the hepatic dysfunction associated with MASLD and T2D may alter their secretion and disrupt metabolic homeostasis [160]. Through their role in communication between the liver and other tissues, hepatokines may contribute to insulin resistance, inflammation, hepatic lipid accumulation, and fibrosis progression. Therefore, they are investigated both as potential biomarkers of MASLD progression and as therapeutic targets for the prevention and treatment of associated metabolic disorders. This chapter presents the main hepatokines for which pharmacological treatments have been investigated, the mechanisms they target, and the available preclinical and clinical evidence regarding their effects in MASLD and T2D.

### 5.1. FGF21 as a Therapeutic Target in MASLD and T2D

FGF21 has gained increasing attention as a therapeutic target because of its role in regulating energy balance, glucose metabolism, and lipid homeostasis through the FGFR1–β-Klotho receptor complex. Although native FGF21 is unsuitable for clinical use, long-acting analogs have shown promising effects on dyslipidemia, hepatic fat content, and liver fibrosis markers, with more limited effects on glycemic control [161].

In preclinical studies on obese mice, FGF21 analogs reduced the expression of lipogenic genes, increased mitochondrial β-oxidation, and produced weight loss, a reduction in hepatic and circulating triglycerides, a decrease in fasting insulinemia and glycemia, as well as an increase in energy consumption [162,163]. In patients with obesity and T2D, FGF21-based treatment improved body weight and lipid profile, particularly by reducing triglycerides and LDL cholesterol and increasing HDL cholesterol, while glucose-lowering effects were limited [164]. Effects on glycemic control and insulin levels have been more variable across studies and did not consistently reach statistical significance [165,166]. FGF21 analogs have also been shown to increase adiponectin levels both in non-human primates and in patients with T2D, obesity, and MASH, suggesting an additional mechanism through which they may improve insulin sensitivity and metabolic homeostasis; however, their overall efficacy remains to be confirmed in large multicenter clinical trials [167,168].

Clinical phase 2b studies in patients with MASH/HASH and F2–F3 fibrosis have reported histological benefits of FGF21 analogs, including ≥1-stage fibrosis improvement and NASH resolution without worsening of fibrosis, particularly with efruxifermin and pegozafermin [169,170]. Efruxifermin, a long-acting subcutaneously administered IgG Fc–FGF21 fusion protein, and pegozafermin, a glycoPEGylated recombinant FGF21 analog administered subcutaneously, are among the most clinically advanced FGF21-based candidates. In patients with MASH and F1–F3 fibrosis, efruxifermin has shown improvements in markers of insulin sensitivity and lipid profile. Although the results obtained so far are promising, the long-term efficacy and safety of FGF21 analogs remain to be confirmed in larger multicenter clinical trials; potential combination strategies with GLP-1 RAs are currently under investigation [171].

### 5.2. Fetuin-A as a Therapeutic Target in MASLD and T2D

Clinical data evaluating the effect of antihyperglycemic drugs on fetuin-A remain limited. In a small study of patients with T2D showed that, although three months of treatment managed to reduce HbA1c in all groups, a significant decrease in fetuin-A occurred only in the group treated with pioglitazone and not in the metformin group [172]. The mechanism was subsequently elucidated in an in vitro study using Fao rat hepatoma cells: pioglitazone, as a PPAR-γ (peroxisome proliferator-activated receptor γ) agonist, directly inhibits hepatic Fetuin-A expression at both the transcriptional and translational levels, an effect blocked by PPAR-γ antagonists, suggesting that this therapeutic class acts specifically through this pathway [173]. A more recent randomized controlled trial (*n* = 88) confirmed that pioglitazone significantly reduces fetuin-A compared with metformin, the reduction being associated with improvement in hepatic steatosis and metabolic inflammation. Nevertheless, a study in insulin-resistant patients with type 1 diabetes (*n* = 40) reported decreases in fetuin-A under metformin therapy as well [174].

Whereas pioglitazone selectively activates PPAR-γ, the pan-PPAR (α/δ/γ) agonist lanifibranor engages the same receptor family more broadly: in a phase 2b trial it significantly improved disease activity and fibrosis in non-cirrhotic NASH, with benefit observed regardless of diabetes status, being in present advancing through phase 3 development [175]. These findings support pan-PPAR agonism as a mechanistically related yet more comprehensive metabolic–antifibrotic approach compared with selective PPAR-γ activation.

Taken together, the data support the hypothesis that the beneficial metabolic effects of pioglitazone are mediated, at least in part, through the modulation of hepatokines, with fetuin-A representing both a relevant biomarker and a potential therapeutic target in the management of patients with T2D and MASLD [173,176,177].

### 5.3. Fetuin-B as a Therapeutic Target in MASLD and T2D

Fetuin-B represents a potential therapeutic target linking MASLD with T2D. In obesity, elevated leptin may stimulate hepatic fetuin-B transcription through signal transducer and activator of transcription 3 (STAT3), and human mediation analyses suggest that fetuin-B partly contributes to leptin-associated insulin resistance [178]. Additionally, apart from its effects on glucose metabolism, fetuin-B appears to drive MASH progression by suppressing adiponectin receptor 1 (AdipoR1) and activating NOD-, LRR- and pyrin domain-containing protein 3 (NLRP3)/gasdermin D (GSDMD)-mediated hepatocyte pyroptosis. Its expression is increased in free fatty acid (FFA)-treated hepatocytes in vitro and high-fat diet-induced MASH mice in vivo, while fetuin-B inhibition reduces hepatic steatosis, inflammation, hepatocyte ballooning, and fibrosis in the mouse model. Together, these preclinical findings identify the fetuin-B-AdipoR1-NLRP3/GSDMD axis as a potential therapeutic target in MASH [179]. However, no pharmacological agents specifically targeting fetuin-B have yet reached clinical development, and its therapeutic potential remains to be validated in humans.

### 5.4. LECT2 as a Therapeutic Target in MASLD and T2D

On a general basis, LECT2 represents a promising therapeutic target due to its role in insulin resistance, hepatic steatosis, and metabolic inflammation. However, no pharmacological agents specifically targeting LECT2 have yet entered clinical development or received regulatory approval. Current evidence is limited to preclinical studies using genetic deletion or RNA-silencing approaches, highlighting the need for further translational research to validate LECT2-targeted therapies in humans [142].

### 5.5. Selenoprotein P as a Therapeutic Target in MASLD and T2D

MASLD has become one of the most prevalent chronic liver diseases worldwide, closely linked to obesity, insulin resistance, T2D, dyslipidemia, and cardiovascular disease [7,180]. Because liver biopsy remains invasive and unsuitable for large-scale screening, there is a growing need for reliable non-invasive biomarkers capable of identifying patients at risk of disease progression and fibrosis [181,182].

Functionally, neutralization of SeP using a monoclonal anti-SeP antibody (AE2) led to improved glucose tolerance, as demonstrated by more efficient glucose clearance following oral glucose administration, together with amelioration of hyperglycemic responses in diabetic mice. In vivo, SeP neutralization improves glucose tolerance and insulin secretion, while enhancing systemic glucose responsiveness in diabetic mouse models. Collectively, these findings support a direct causal role of SeP in impairing glucose metabolism in diabetic mouse models and demonstrate that its inhibition can reverse key pathological features of diabetes in vivo [183].

Selenoprotein P thus represents a promising therapeutic goal because of its role in insulin resistance and glucose dysregulation. To date, SeP-neutralizing monoclonal antibodies constitute the only specific therapeutic instrument evaluated in preclinical studies, whereas no SeP-targeted therapies have yet entered clinical development or received regulatory approval.

### 5.6. ANGPTL3 and ANGPTL8 as Therapeutic Targets in MASLD and T2D

Therapeutic targeting of ANGPTL3 has been explored through multiple pharmacological modalities. Among these, monoclonal antibody-based inhibition, exemplified by evinacumab, has proved clinically meaningful reductions in triglycerides and LDL cholesterol, also in patients with severe and treatment-refractory hypercholesterolemia. Importantly, these lipid-lowering effects appear to occur independently of LDL receptor function, suggesting potential utility in a broader range of dyslipidemic phenotypes. In parallel, nucleic acid-based strategies such as antisense oligonucleotides and RNA interference therapies are under investigation and have further reinforced the validity of ANGPTL3 as a drug-related target [184].

While most actual clinical evidence originates from studies within familial hypercholesterolemia and mixed dyslipidemia, the mechanistic role of ANGPTL3 in triglyceride metabolism and insulin-resistant states provides a rationale for its potential relevance in T2D–associated dyslipidemia. Nevertheless, long-term data regarding cardiovascular outcomes and metabolic benefits in this population remain limited, and ongoing studies are expected to better define its place in future lipid-lowering strategies [185,186].

Among nucleic acid-based therapies, vupanorsen (AKCEA-ANGPTL3-LRx), a GalNAc-conjugated antisense oligonucleotide targeting hepatic ANGPTL3 mRNA, was the first agent to reach phase 2 clinical development and demonstrated significant reductions in circulating ANGPTL3, triglycerides, and non-HDL cholesterol. However, its clinical development was discontinued because of dose-dependent increases in hepatic fat content and liver enzyme abnormalities [187]. More recently, liver-targeted small interfering RNA (siRNA) therapies have proved a more favorable efficacy and safety profile. Zodasiran (ARO-ANG3) and solbinsiran (LY3561774), both designed to silence hepatic ANGPTL3 expression, have produced sustained reductions in circulating ANGPTL3, triglycerides, LDL cholesterol, apolipoprotein B and remnant cholesterol, in patients with mixed dyslipidemia [188,189]. In the phase 2 PROLONG-ANG3 trial, solbinsiran maintained lipid-lowering effects for several months after only two subcutaneous administrations and was generally well tolerated, supporting RNA interference as a convenient therapeutic strategy for long-term ANGPTL3 inhibition [190].

### 5.7. RBP4 as a Potential Biomarker

Preclinical evidence further supports the concept that lowering RBP4 may improve metabolic dysfunction. Long-term fenretinide treatment partially prevented or reversed obesity, insulin resistance, and hepatic steatosis in high-fat diet-fed mice, suggesting that modulation of retinoid/RBP4 metabolism may influence both glucose homeostasis and liver fat accumulation [191]. Similarly, RNA oligonucleotide against RBP4 ameliorated hepatic steatosis in mice [192]. Another indirect strategy involves reducing circulating transthyretin (TTR) with antisense oligonucleotides, which decrease TTR and RBP4 levels, improving insulin sensitivity and glucose metabolism in obese mice, without body weight shift [193].

## 6. Antidiabetic Therapies and Their Impact on Hepatokines

T2D is closely linked to MASLD, and this bidirectional relationship has increased the interest in the potential liver-related effects of antidiabetic therapies [194]. Metformin, the first-line treatment of T2D, may improve hepatic steatosis, while newer agents, including GLP-1 RAs and SGLT2i, have shown cardiovascular and hepatic benefits [195,196]. Moreover, these benefits may extend beyond glycemic control; some antidiabetic therapies may also modulate circulating hepatokines involved in insulin resistance and metabolic homeostasis. This chapter addresses the impact of the most commonly used antidiabetic drugs on hepatokines, highlighting the available evidence and the inconsistencies that remain.

### 6.1. Metformin

Metformin is an oral hypoglycemic agent with a remarkable history, a very favorable safety profile, proven efficacy and low cost; for precisely these reasons, it has become and remained the first-line drug in the treatment of T2D worldwide, recommended by international guidelines and societies [197]. The main target organ for metformin is the liver, while its central effect relies on the inhibition of hepatic glucose production. However, its mechanism of action is not yet fully understood and appears to involve several dose- and time-dependent pathways, including AMPK-dependent and AMPK-independent mechanisms, mitochondrial and lysosomal targets, and effects on the gut, gut microbiota, and immune system [198,199].

In the context of the frequent overlap between T2D and MASLD, metformin is of particular therapeutic interest. By reducing insulin resistance and hyperglycemia, metformin attenuates two of the principal pathogenic factors of MASLD (7). Preclinical studies have demonstrated significant anti-inflammatory and antifibrotic effects: metformin inhibits the activation of hepatic stellate cells, suppresses transforming growth factor beta (TGF-β)-dependent pro-fibrotic pathways, and reduces the expression of fibrosis markers [200,201]. Through AMPK activation, it suppresses hepatic de novo lipogenesis, stimulates fatty acid β-oxidation, and reduces intracellular oxidative stress. Epidemiological data also suggest that metformin may lower the risk of hepatocellular carcinoma in individuals with diabetes, with one of the most substantial reductions in incidence reported among cancers [202]. Its anti-inflammatory effects also include modulation of the gut–liver axis, influencing the composition of the intestinal microbiome, thus reducing the MASLD-associated dysbiosis and increased intestinal permeability [200].

The effects of metformin also extend to hepatokines, such as FGF21, although they appear to vary across experimental and clinical settings. In primary rat and human hepatocytes, metformin increased FGF21 expression in a dose-dependent manner through an AMPK-dependent mechanism, suggesting that hepatic FGF21 induction may contribute to its antidiabetic effects [203]. In contrast, another study conducted on cell lines, mice or human subjects reported that metformin increased hepatic and circulating FGF21 through an AMPK-independent mechanism involving mitochondrial complex I inhibition and activation of the PERK-eIF2α-ATF4 pathway [204]. Clinical findings have also been inconsistent. In patients with newly diagnosed T2D, circulating FGF21 levels were elevated compared with normoglycemic controls but decreased after 12 weeks of metformin therapy, possibly reflecting improvements in glucose–lipid metabolism and systemic inflammation [205]. In a separate 12-week randomized controlled trial, metformin did not alter circulating FGF21 levels but reduced fibroblast activation protein (FAP) activity and increased adipose expression of FGFR1c and β-klotho, suggesting that it may enhance peripheral FGF21 sensitivity rather than FGF21 secretion [206]. Similarly, short-term metformin treatment in healthy individuals did not affect FGF21 levels, although it increased growth differentiation factor 15 (GDF15), with in vitro findings indicating that the gastrointestinal tract may be an important source of metformin-induced GDF15 [207].

On fetuins, metformin may have variable effects, depending on different metabolic conditions. In patients with newly diagnosed T2D, 12 weeks of metformin reduced osteoprotegerin levels only in women, without a significant effect on fetuin-A, while the observed changes were not clearly associated with improvements in insulin resistance [177]. However, in patients with T2D and MASLD, 120 days of metformin reduced circulating fetuin-A levels and improved vibration-controlled transient elastography (VTE) parameters, without significantly changing hepatic or pancreatic fat fractions assessed by MRI-PDFF [174]. In adolescent girls with PCOS, a 12-month treatment regimen combining metformin, spironolactone, and pioglitazone, but no oral contraception, normalized fetuin-A levels and reduced hepatic fat, although the specific contribution of metformin could not be determined [208]. On the other hand, in women with PCOS, 24 weeks of metformin therapy was associated with a significant reduction in circulating fetuin-B levels and improvements in metabolic parameters, suggesting that fetuin-B may serve as a potential biomarker of treatment response [209].

Preclinical studies suggest that metformin suppresses hepatic SeP production through several mechanisms. In hepatocytes and mouse liver, this effect involved AMPK-mediated phosphorylation and inactivation of protein Forkhead box O3a (FoxO3a) [210], while in rat hepatocytes, metformin dose-dependently suppressed SeP production and reduced selenophosphate synthetase 2 expression [211]. However, in patients with poorly controlled T2D, 12 weeks of metformin did not significantly change circulating SeP levels, but higher baseline concentrations predicted greater improvements in glycemic control and insulin secretion, suggesting that SeP may serve as a biomarker of treatment response [212]. Metformin also reduced ANGPTL3 expression in HepG2 cells, through an AMPK–SIRT1-independent mechanism, potentially contributing to the regulation of lipoprotein lipase activity and lowering plasma lipid levels [213].

### 6.2. SGLT2 Inhibitors

SGLT2i represent a distinct class with their individual hepatic mechanisms, more complex than initially considered. These agents ameliorate hepatic steatosis by promoting fatty acid β-oxidation and inhibiting lipogenesis, improve insulin sensitivity, and attenuate inflammation, oxidative stress, and fibrosis. Clinically, the use of SGLT2i is associated with a reduction in liver enzymes, improvement in histological features, and potential suppression of hepatocarcinogenesis, with benefits observed in both patients with and without T2D [214,215,216].

Amin et al. investigated the effect of empagliflozin on hepatic steatosis in patients with T2D and MASLD using MRI-derived proton density fat fraction (MRI-PDFF). After 24 weeks of treatment, empagliflozin significantly reduced liver fat content by approximately 13.2%, accompanied by significant decreases in body weight, body mass index, fasting blood glucose, and ALT levels. However, no significant improvements were observed in non-invasive fibrosis markers, including the Fibrosis-4 (FIB-4) index and MASLD Fibrosis Score, suggesting that the short-term benefits of SGLT2 inhibition are more pronounced for hepatic steatosis than for liver fibrosis [217].

Regarding hepatokines, studies focusing on the effects of SGLT2i on FGF21 levels have also yielded inconsistent results. In the EFFECT-II trial, dapagliflozin monotherapy significantly reduced circulating FGF21 levels, liver fat content, and markers of hepatocellular injury, although the change in FGF21 was not associated with the reduction in liver fat [218]. Two smaller studies likewise found no significant overall change in circulating FGF21 after dapagliflozin treatment, despite improvements in body weight and glycemic control. However, one of these studies reported that changes in FGF21 were positively correlated with changes in body weight [219,220].

In patients with T2D and MASLD, 120 days of dapagliflozin treatment reduced circulating fetuin-A levels alongside improvements in body weight, BMI, and percentage body fat [174]. By contrast, in a randomized study of patients with early-stage T2D and MASLD, empagliflozin reduced intrahepatic lipid content more effectively than sitagliptin, while fetuin-A levels remained unchanged in both groups, suggesting that the reduction in liver fat was not associated with changes in circulating fetuin-A [221].

### 6.3. GLP-1 Receptor Agonists

GLP-1 is an incretin hormone with important physiological roles, including stimulation of glucose-dependent insulin secretion, delayed gastric emptying, and promotion of satiety [222]. Exogenous administration of GLP-1 to patients with T2D restores insulin secretion, suppresses glucagon, normalizes glycemia, and leads to a decrease in free fatty acid concentrations, reduced appetite, and improved insulin sensitivity, with a net favorable impact on metabolic control and body weight. Because native GLP-1 has an extremely short half-life, GLP-1 RAs with increased molecular stability and prolonged duration of action have been synthesized, designed to reproduce and magnify the endogenous hormone effects [223].

In vitro studies using human hepatocytes suggest that GLP-1 RAs may exert direct hepatoprotective effects by activating GLP-1 receptor signaling, thereby reducing intracellular triglyceride accumulation, enhancing autophagy, attenuating endoplasmic reticulum stress, and protecting hepatocytes against fatty acid-induced apoptosis [224,225]. However, a direct GLP-1 receptor-mediated effect at the hepatocyte level remains controversial, as more recent evidence has not confirmed functional GLP-1 receptor signaling in primary human hepatocytes [226]. Clinical evidence has been reported for several agents in this therapeutic class. Pemvidutide, a dual GLP-1/glucagon receptor agonist, produced a significant reduction in liver fat content, body weight, and hepatic inflammation markers in MASLD patients compared to placebo [227]. Semaglutide demonstrated dose-dependent efficacy in promoting steatohepatitis resolution in a phase 2 trial [228], while the phase 3 ESSENCE trial confirmed that semaglutide 2.4 mg once weekly significantly increased both MASH resolution without worsening of fibrosis and fibrosis improvement without worsening of MASH compared with placebo [229]. In addition, liraglutide promoted histological resolution of non-alcoholic steatohepatitis and reduced the proportion of patients experiencing fibrosis progression in the LEAN (Safety and Efficacy in Patients with Non-Alcoholic Steatohepatitis) trial [230].

Given the beneficial effects of GLP-1 RAs on the progression of MASLD and T2D, recent studies have investigated their impact on hepatokines with the aim of elucidating the mechanisms underlying their therapeutic effects. In this context, in a 24-week randomized trial of patients with T2D and NAFLD, liraglutide produced a greater reduction in circulating fetuin-A levels than pioglitazone and was also associated with a significant decrease in hepatic fat content. The decline in fetuin-A correlated with reductions in body weight, BMI, waist circumference, and liver fat, suggesting that the hepatic benefits of liraglutide may be driven largely by weight loss rather than by improved glycemic control alone [231]. Similarly, six months of GLP-1 RA therapy significantly reduced fetuin-B concentrations in parallel with improvements in insulin sensitivity in women with PCOS [232]. In patients with T2D, exenatide increased circulating ANGPTL8 levels despite improvements in body weight and glycemic control, while in vitro findings in HepG2 cells indicated that GLP-1 RAs directly stimulate hepatic ANGPTL8 production through a GLP-1R-dependent PI3K/Akt pathway [233].

Several studies suggest that FGF21 may contribute to the beneficial hepatic effects of GLP-1 RAs, although the relationship is complex. Preclinical studies using diabetic or diet-challenged mouse models and primary mouse hepatocytes indicate that agents such as liraglutide, exenatide, and semaglutide can stimulate hepatic FGF21 expression or improve FGF21 responsiveness, thereby supporting reductions in hepatic glucose production and lipid accumulation [234,235,236]. However, findings in humans are less consistent. Some clinical studies have reported a decrease in circulating FGF21 after treatment with exenatide or liraglutide, often in parallel with improvements in insulin resistance and liver fat content, whereas others have observed an increase or no significant change [158,237,238]. These differences may reflect variations in treatment duration, patient characteristics, disease severity, and the distinction between hepatic FGF21 production and circulation FGF21 concentrations.

## 7. Future Perspectives

Despite the growing body of evidence supporting the involvement of hepatokines in the pathophysiology of MASLD and T2D, several important challenges remain before these molecules can be translated into routine clinical practice. Most current data come from cross-sectional studies, experimental models, or early-phase clinical trials, and prospective longitudinal studies validating the diagnostic and prognostic value of hepatokines across different stages of MASLD and T2D are still limited. Future research should move beyond the evaluation of individual hepatokines and instead examine their complex interplay with adipokines, myokines, gut-derived mediators, and inflammatory pathways, which together regulate systemic metabolic homeostasis. In this regard, multi-omics technologies, integrated biomarker panels, and artificial intelligence-based predictive models could help improve disease stratification and identify patients at increased risk of progressive liver disease, diabetes, or cardiometabolic complications.

From a therapeutic perspective, hepatokine-targeted interventions represent an emerging area of translational research. FGF21 analogs are the most clinically advanced so far, whereas strategies targeting fetuin-A, fetuin-B, LECT2, SeP, ANGPTLs, and RBP4 remain earlier in development and will require further preclinical and clinical validation. Understanding how established metabolic therapies, including GLP-1 RAs, SGLT2 inhibitors, and combined treatment strategies, modulate hepatokine signaling could also help guide more individualized therapeutic approaches. Ultimately, integrating hepatokine profiling into precision medicine frameworks may improve earlier diagnosis and personalized management of patients with MASLD and T2D.

## 8. Conclusions

While earlier reviews have addressed the role of hepatokines in MASLD, insulin resistance, and T2D, most have focused on either hepatic disease progression or broader systemic metabolic dysfunction. This review, in contrast, offers an integrated perspective on the bidirectional relationship between MASLD and T2D, positioning hepatokines as central mediators of liver–organ crosstalk and highlighting their pathophysiological, diagnostic, and therapeutic relevance. The available data indicate that hepatokines, including FGF21, fetuin-A, fetuin-B, LECT2, SeP, ANGPTLs, and RBP4, not only reflect metabolic dysfunction but actively contribute to insulin resistance, hepatic steatosis, inflammation, fibrosis, and disease progression. Moreover, growing evidence suggests that modulating hepatokine signaling, whether through emerging targeted therapies or currently available antidiabetic agents, may represent a promising strategy for improving both hepatic and metabolic outcomes.

## Figures and Tables

**Table 1:** Major hepatokines linking MASLD and type 2 diabetes.

Hepatokine	General Characteristics	Role in MASLD/MASH	Role in T2D	Source
FGF21	Mainly produced by the liver. Acts through FGF receptors with the co-receptor β-klotho.	Generally hepatoprotective. Reduces hepatic triglyceride accumulation, promotes fatty acid oxidation, suppresses de novo lipogenesis, improves mitochondrial function, reduces oxidative stress and inflammation, and may attenuate fibrosis progression.	May improve insulin sensitivity by reducing glucotoxicity and lipid spillover. However, circulating levels are often elevated in obesity, insulin resistance, and T2D, suggesting possible “FGF21 resistance”.	[44,45,46,47,48,49,50,51,52,53]
Fetuin-A	Mainly secreted by the liver, with extrahepatic sources including visceral and subcutaneous adipose tissue.	Fetuin-A can worsen hepatic and systemic insulin resistance, increase free fatty acid delivery to the liver, activate TLR4/NF-κB signaling, and contribute to inflammation, steatohepatitis, and fibrosis.	Inhibits insulin receptor signaling and GLUT4 translocation, reducing peripheral glucose uptake. Associated with inflammation, insulin resistance, impaired glucose tolerance, and higher T2D risk.	[54,55,56,57,58,59,60,61,62,63,64,65,66,67]
Fetuin-B	Liver-derived glycoprotein mainly produced by hepatocytes and structurally similar to fetuin-A.	Increased in hepatic lipid accumulation and MASLD. Associated with hepatic steatosis and MASLD-related metabolic dysfunction.	Impairs insulin action in hepatocytes and myotubes, induces glucose intolerance, and may contribute to peripheral insulin resistance through liver–adipose tissue crosstalk.	[39,42,68,69,70,71,72,73]
LECT2	Multifunctional secreted protein mainly produced by hepatocytes. Initially described as a neutrophil chemotactic factor	Linked to hepatic lipid accumulation, inflammation, and metabolic dysfunction. Increased in steatotic and inflamed liver tissue. May promote inflammation through macrophage polarization	Higher circulating levels reported in newly diagnosed T2D, obesity, and metabolic syndrome. Associated with HOMA-IR, fasting insulin, BMI, triglycerides, HDL-C, adipose tissue dysfunction, and insulin resistance.	[74,75,76,77,78,79,80,81,82,83,84,85,86,87,88]
Selenoprotein P	Main selenium transport protein in mammals	Linked to hepatic steatosis, insulin resistance, inflammation, and fibrosis, although evidence is partly conflicting. Some studies report higher SeP in MASLD, while others report lower levels in definite NASH.	Higher levels reported in T2D or prediabetes. Associated with BMI, waist circumference, triglycerides, glucose, HbA1c, inflammatory markers, and insulin resistance.	[82,89,90,91,92,93,94,95,96,97,98,99,100,101]
ANGPTL8	Angiopoietin-like proteins involved in the regulation of triglyceride metabolism	Updated meta-analytic evidence continues to support higher circulating ANGPTL8 levels in steatotic liver disease related to metabolic dysfunction, but heterogeneity remains high	ANGPTL8 is elevated in obesity, T2D, gestational diabetes mellitus, and polycystic ovary syndrome, especially in relation to insulin resistance and metabolic dysfunction.	[102,103,104,105,106,107,108,109,110,111,112,113,114,115,116]
RBP4	Secreted predominantly by the liver and adipose tissue. It was originally identified as the main circulating transport protein for retinol and other retinoid derivatives	Higher circulating RBP4 is associated with MASLD, hepatic steatosis, obesity, insulin resistance, and disease severity, suggesting potential value as a non-invasive biomarker, although causality is not established.	Elevated RBP4 is linked to insulin resistance, impaired glucose metabolism, and increased T2D risk. It may impair insulin signaling in metabolic tissues and contribute to β-cell dysfunction through the STRA6–JAK2/STAT1 pathway.	[117,118,119,120,121,122]

## Data Availability

No new data were created or analyzed in this study. Data sharing is not applicable to this article.
